# Enhanced Antiproliferative Effect of Combined Treatment with Calcitriol and All-*Trans* Retinoic Acid in Relation to Vitamin D Receptor and Retinoic Acid Receptor α Expression in Osteosarcoma Cell Lines

**DOI:** 10.3390/ijms21186591

**Published:** 2020-09-09

**Authors:** Silvia Paukovcekova, Dalibor Valik, Jaroslav Sterba, Renata Veselska

**Affiliations:** 1 Regional Centre for Applied Molecular Oncology, Masaryk Memorial Cancer Institute, Zluty kopec 7, 65653 Brno, Czech Republic; silvia.paukovcekova@mail.muni.cz (S.P.); valik@mou.cz (D.V.); 2 Laboratory of Tumor Biology, Department of Experimental Biology, Faculty of Science, Masaryk University, Kotlarska 2, 61137 Brno, Czech Republic; 3 Department of Pediatric Oncology, University Hospital Brno and Faculty of Medicine, Masaryk University, Cernopolni 9, 61300 Brno, Czech Republic; Sterba.Jaroslav@fnbrno.cz; 4 International Clinical Research Center, St. Anne’s University Hospital Brno, Pekarska 53, 65691 Brno, Czech Republic

**Keywords:** osteosarcoma, calcitriol, calcidiol, all-*trans* retinoic acid, vitamin D receptor, retinoic acid receptor α

## Abstract

The main objective of this study was to analyze changes in the antiproliferative effect of vitamin D3, in the form of calcitriol and calcidiol, via its combined application with all-*trans* retinoic acid (ATRA) in osteosarcoma cell lines. The response to treatment with calcitriol and calcidiol alone was specific for each cell line. Nevertheless, we observed an enhanced effect of combined treatment with ATRA and calcitriol in the majority of the cell lines. Although the levels of respective nuclear receptors did not correlate with the sensitivity of cells to these drugs, vitamin D receptor (VDR) upregulation induced by ATRA was found in cell lines that were the most sensitive to the combined treatment. In addition, all these cell lines showed high endogenous levels of retinoic acid receptor α (RARα). Our study confirmed that the combination of calcitriol and ATRA can achieve enhanced antiproliferative effects in human osteosarcoma cell lines in vitro. Moreover, we provide the first evidence that ATRA is able to upregulate VDR expression in human osteosarcoma cells. According to our results, the endogenous levels of RARα and VDR could be used as a predictor of possible synergy between ATRA and calcitriol in osteosarcoma cells.

## 1. Introduction

Osteosarcoma is a high-grade primary mesenchymal tumor characterized by spindle cells depositing an immature osteoid matrix [1]. To date, osteosarcoma is the most frequent primary malignancy of bone in children and the most frequent primary malignancy in adolescents apart from leukemia and lymphoma [2,3]. Surgical excision is often effective only for patients with low-grade tumors [4]. For patients with high-grade tumors, other therapeutic methods, such as chemotherapy and radiotherapy, must also be employed [5]. Chemotherapy used in osteosarcoma protocols remains essentially unchanged since the introduction of high-dose methotrexate, doxorubicin, and cisplatin in the late 1970s [6,7,8]. The five-year overall survival has remained approximately 60% over the last five decades; nevertheless, the overall survival of patients with metastatic osteosarcoma is <20% [9]. Multiple efforts to improve therapeutic efficacy have not identified more effective or less toxic regimens, despite intensifying treatment or modulating the immune response [7,10,11,12]. Therefore, new therapeutic approaches are urgently needed.

Induced differentiation of transformed cells into mature phenotypes has proven to be an effective strategy in the treatment of several types of human malignancies [13,14], and derivatives of vitamin A, retinoids, are some of the most frequently used inducers of differentiation [15,16,17,18]. The molecular mechanism of retinoid signaling is based on their binding to members of the nuclear receptor family, retinoic acid receptor (RAR) and retinoid X receptor (RXR), which subsequently form homodimers or heterodimers, bind to the DNA, and influence transcription directly, or they can interact with other transcription factors. In addition to their nuclear transcriptional effects, retinoids are able to rapidly and transiently activate several kinase signaling pathways [19].

Despite the many benefits of retinoids as anticancer compounds, their usage in clinical protocols is still limited because of their short intracellular availability, clinically significant toxicity, and the occurrence of resistance [20]. Therefore, efforts have been made to include retinoids in combined treatment with other drugs that may enhance or prolong their antineoplastic effects. Combinations of all-*trans* retinoic acid (ATRA) with several natural compounds, kinase inhibitors, chemotherapeutics, and proteasome inhibitors have demonstrated additive or synergistic effects [21]. Our research group described the enhancement of the antineoplastic effect of ATRA caused by inhibition of its catabolism using LOX/COX inhibitors (caffeic acid and celecoxib) in neuroblastoma, medulloblastoma, and osteosarcoma cell lines [22,23,24,25]. The benefits of combined treatment in the therapy of several solid tumors have also been confirmed for retinoic acid and other differentiation inducers, such as calcitriol [26,27,28].

Calcitriol (1α,25(OH)_2_ vitamin D_3_) is the most biologically active form of vitamin D_3_ [29]. It is mainly synthesized endogenously via UVB radiation of human skin followed by stepwise hydroxylation in the liver and kidney or can be obtained by exogenous dietary intake [30,31]. In animal cells, calcitriol binds to the nuclear vitamin D receptor (VDR), which is subsequently transported to the nucleus, where it forms dimers. The dimer complex acts as a transcription factor that can either activate or suppress mRNA expression after binding to the vitamin D responsive element in the promotor region of several target genes that are primarily involved in the calcium homeostasis of cell differentiation, in bone formation, resorption, and mineralization, and in the maintenance of neuromuscular function [32]. The recent meta-analysis suggests that calcitriol and its precursor calcidiol (25(OH) vitamin D_3_) could act as chemopreventive agents [33]. The correlations between low serum levels of calcidiol and increased mortality of patients with colorectal cancer [34], prostate cancer [35], breast cancer [36] and melanoma [37] have also been reported. To date, several studies on the antineoplastic effects of calcitriol in osteosarcoma have been published [38,39,40,41,42,43]. Nevertheless, the dose-dependent response to calcitriol and calcidiol in different osteosarcoma cell lines is still not well defined, and the mechanisms involving the inhibition of proliferation and differentiation induction remain unclear [44].

In the present study, we focused on the possible effects of calcitriol and calcidiol alone or in combination with ATRA in patient-derived osteosarcoma cell lines, with special regard to the mechanism of interaction between calcitriol and ATRA.

## 2. Results

### 2.1. Calcitriol Slightly Increases the Antiproliferative Effect of ATRA in the Saos-2 Reference Cell Line

**Figure 1:**
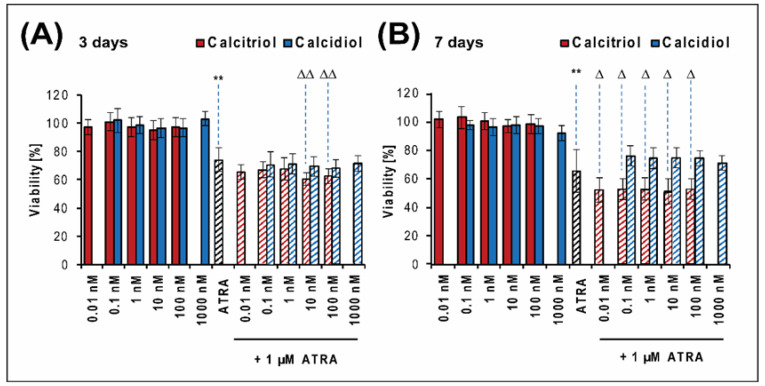
Proliferation of the Saos-2 cell line after 3 (**A**) and 7 (**B**) days of selected treatment. The proliferation was measured using the MTT assay on days 3 and 7 of incubation with various concentrations of calcitriol or calcidiol alone, 1 μM all-*trans* retinoic acid (ATRA) alone, or drug combinations. The values were compared with those in untreated cells, whose proliferation activity was set at 100%. The data represent the mean ± SD. The results were analyzed using one-way ANOVA, followed by the Scheffé post hoc test. The proliferation of cells treated with calcitriol or calcidiol alone was compared to the proliferation of untreated control cells: ** *p* < 0.001. The proliferation of cells treated with a combination of drugs was compared to the proliferation of cells treated with ATRA alone: ∆ < 0.05, ∆∆ < 0.001. Experiments were performed in biological triplicate.

First, we investigated the possible effects of calcidiol and calcitriol—either alone or in combination with ATRA—on the Saos-2 established cell line. Using the MTT assay, an analysis of cell viability was performed on days 3 (Figure 1A) and 7 (Figure 1B) of the selected treatment. No apparent reduction in cell viability after treatment with calcidiol or calcitriol alone was observed (Figure 1). The chosen concentration of calcidiol (100 nM) and calcitriol (10 nM) had no effect on these cells at all.

The combined treatment of Saos-2 cells with ATRA and calcitriol enhanced synergistically the inhibitory effect of ATRA alone (Table 1). The detailed calculations of possible interactions are provided in Table A1 and Table A2. On day 3, ATRA in combination with 10 nM and 100 nM calcitriol significantly decreased the viability in comparison with ATRA alone (Figure 1A). At day 7, all combinations of calcitriol and ATRA significantly enhanced the inhibitory effect of ATRA (Figure 1B). Calcidiol in combination with ATRA had a slightly antagonistic effect compared with ATRA alone (Figure 1B, Table 1).

**Figure 2:**
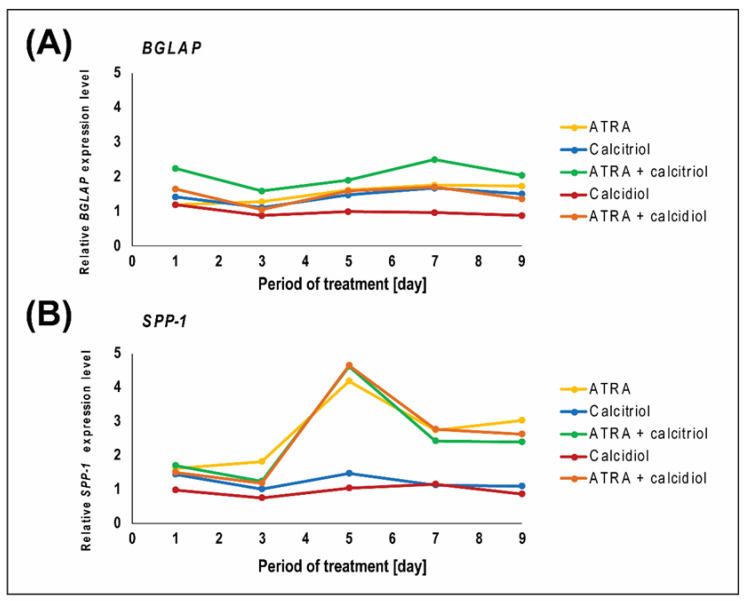
Expression of markers of osteogenic differentiation in the Saos-2 cell line. Data represent the evaluation of the mRNA expression of *BGLAP* (**A**) and *SPP1* (**B**). Cells were treated with 10 nM calcitriol, 100 nM calcidiol, 1 μM ATRA, or drug combinations. The results were obtained on days 1, 3, 5, 7, and 9 of treatment using semiquantitative RT-PCR. The expression levels were quantified in ImageJ using densitometry. *HSP90AB1* served as a loading control. The levels of gene expression after drug treatment were compared to the levels detected in untreated control samples (value y = 1). Data represent the mean. Experiments were performed in biological triplicate. Additional statistical data are provided in Table A3.

The expression of the *BGLAP* and *SPP1* genes, which encode the markers of osteogenic differentiation osteocalcin and osteopontin, respectively, was evaluated using RT-PCR. The changes in the expression of these genes during nine days of treatment are shown in Figure 2. Additional statistical data are provided in Table A3. Overall, calcidiol had no effect on *BGLAP* expression, whereas calcitriol was able to upregulate *BGLAP* expression from the first day of treatment (Figure 2A). Neither calcitriol nor calcidiol caused any significant upregulation of *SPP1* (Figure 2B). The highest increase in *BGLAP* expression during the entire test period was caused by the combination of ATRA and calcitriol, which was markedly stronger than the effect caused by ATRA alone (Figure 2A). The synergy between ATRA and calcitriol action was observed at days 1, 3, and 7 (Table 2). In contrast, the effect of all drug combinations on *SPP1* expression was comparable to the effect of ATRA alone (Figure 2B) and the antagonism was also identified (Table 2).

### 2.2. Patient-Derived Osteosarcoma Cell Lines Show Various Levels of Sensitivity to Calcitriol, Calcidiol, and Their Combinations with ATRA

In general, all six patient-derived cell lines included in this study showed higher sensitivity to calcidiol or calcitriol alone than the Saos-2 established cell line. Nevertheless, the responsiveness of these cell lines to the experimental treatment varied.

At day 3 of the treatment, neither the drugs alone nor their combinations affected the cell viability, except for OSA-02 and OSA-09 cell lines, which were sensitive to the treatment with 100 nM calcitriol (Supplement 1).

**Figure 3:**
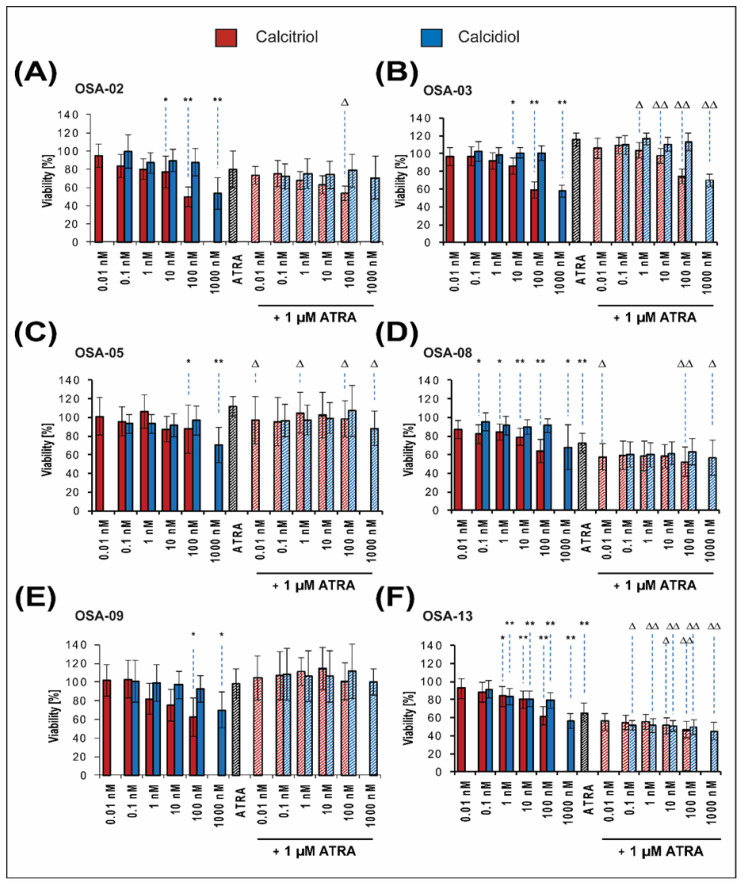
Proliferation of patient-derived osteosarcoma cell lines after 7 days of selected treatment. The proliferation of the OSA-02 (**A**), OSA-03 (**B**), OSA-05 (**C**), OSA-08 (**D**), OSA-09 (**E**), and OSA-13 (**F**) cell lines was measured using the MTT assay on day 7 of incubation with various concentrations of calcitriol or calcidiol alone, 1 μM ATRA alone, or drug combinations. The values were compared with those of untreated cells, whose proliferation activity was set at 100%. The data represent the mean ± SD. The results were analyzed using one-way ANOVA, followed by the Scheffé post hoc test. The proliferation of cells treated with calcitriol or calcidiol alone was compared to the proliferation of untreated control cells: * *p* < 0.05, ** *p* < 0.001. The proliferation of cells treated with a combination of drugs was compared to the proliferation of cells treated with ATRA alone: ∆ < 0.05, ∆∆ < 0.001. Experiments were performed in biological triplicate.

At day 7 of the treatment, a dose-dependent decrease in cell viability with an increasing concentration of calcitriol was obvious in all cell lines (Figure 3). Calcitriol treatment at all concentrations reduced the viability of the cell lines by up to 50% of the respective control value: OSA-02 cells were identified as the most sensitive to the treatment with 100 nM calcitriol (Figure 3A). Calcitriol at concentration of 10 nM significantly reduced the viability of OSA-02, OSA-03, OSA-08, and OSA-13 cell lines (Figure 3A,B,D,F). The OSA-13 cell line was identified as the most sensitive to the treatment with calcidiol; a significant reduction in viability was observed at a 1 nM concentration (Figure 3F). In all other patient-derived cell lines, a significant response was induced by treatment with 1 µM calcidiol (Figure 3A–E).

At day 7, the cell viability after treatment with ATRA varied between 60% and 100% in comparison with that of untreated control cells. The combined treatment with ATRA and calcitriol significantly enhanced the effect of ATRA alone in five cell lines (Figure 3A–D,F). In these cell lines, the combined effects of drugs evaluated using the Bliss independence model were predominantly identified as additive (Table 3). In contrast, no effect was found after the same combined treatment in the OSA-09 cell line when compared with the effect of ATRA alone (Figure 3E). Nevertheless, the combined effect of ATRA and calcitriol was identified as antagonistic in OSA-09 cells (Table 3). Calcidiol at any concentration did not significantly affect the action of ATRA in OSA-02 and OSA-09 cell lines (Figure 3A,E), but it was able to enhance the effect of ATRA alone in the OSA-13 cell line (Figure 3F). A significantly stronger effect of combined treatment with 1 µM calcidiol and ATRA was observed in OSA-03, OSA-05, and OSA-08 cell lines (Figure 3B–D). Using the Bliss model, the synergy or the additive effects between ATRA and calcidiol actions were detected in OSA-03, OSA-05, OSA-08, and OSA-13 cells (Table 4). In OSA-02 and OSA-09 cells, the effects of such combined treatment were identified predominantly as antagonistic (Table 4).

### 2.3. ATRA Influences VDR Expression

Based on the variable sensitivity of patient-derived cell lines to the experimental treatment, as described above, in the next step, we aimed to analyze the expression of receptors for these drugs in untreated cells. Therefore, we focused on the expression of vitamin D receptor (VDR), retinoic acid receptor (RARα), and retinoid X receptor (RXRα), which is a dimerization partner for both RARα and VDR.

**Figure 4:**
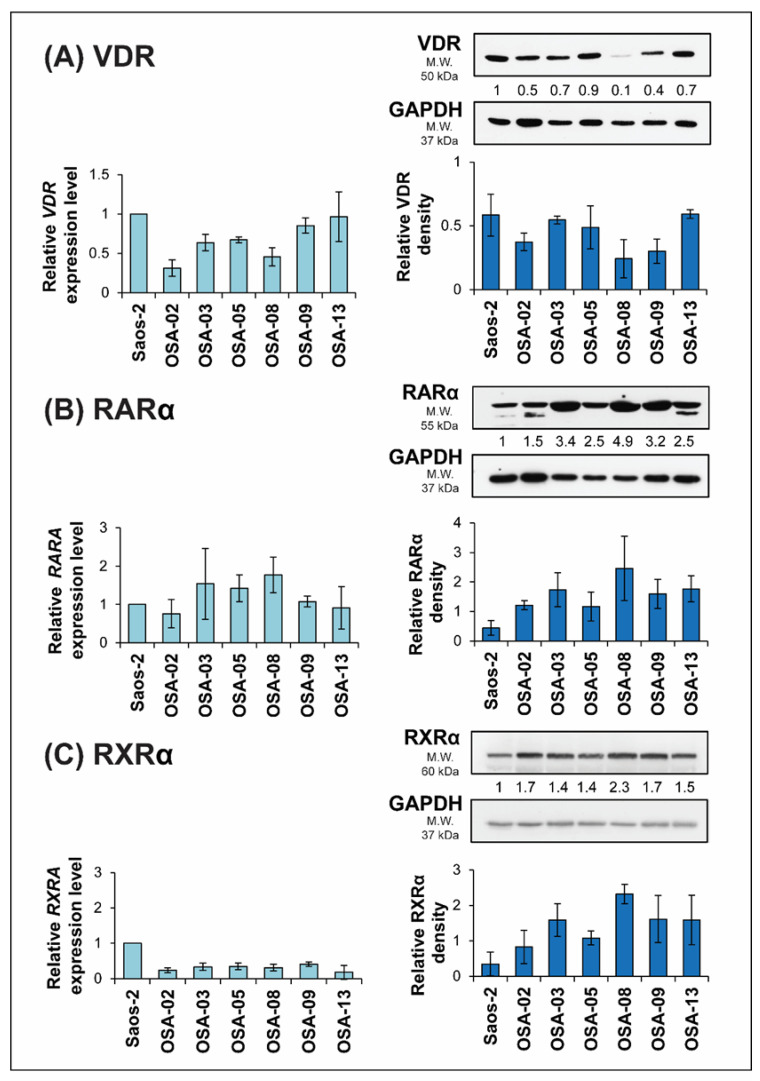
Expression of VDR (**A**), RARα (**B**), and RXRα (**C**) at both the mRNA and protein levels in untreated osteosarcoma cell lines. The relative expression of selected genes was measured using RT-qPCR, and the mRNA levels are presented as fold changes compared to the levels detected in the Saos-2 reference osteosarcoma cell line, which served as an arbitrary calibrator. The levels of selected proteins were measured by immunoblotting. Densitometry of protein bands was performed using Image J software and was represented as the ratio of respective receptor level (VDR, RARα, and RXRα) to level of loading control (GAPDH). The data represent the mean ± SD. Experiments were performed in biological triplicate.

Low levels of *VDR* mRNA were found in OSA-02 and OSA-08 cell lines, with high levels found in the Saos-2 reference cell line, as well as in the OSA-09 and OSA-13 cell lines (Figure 4A). In contrast, the expression of *RARA* showed an almost inverse pattern to *VDR* expression (Figure 4B). The highest level of *RXRA* mRNA was found in the Saos-2 cell line; all patient-derived cell lines showed markedly decreased *RXRA* expression (Figure 4C).

For the VDR and RARα receptors, the results from immunoblotting were in accordance with those from qPCR: the inverse patterns of these proteins were also apparent (Figure 4A,B). Surprisingly, RXRα protein levels did not correlate with gene expression. Although the highest levels of *RXRA* mRNA were detected in Saos-2 cells, the protein level was markedly reduced in this cell line (Figure 4C).

**Figure 5:**
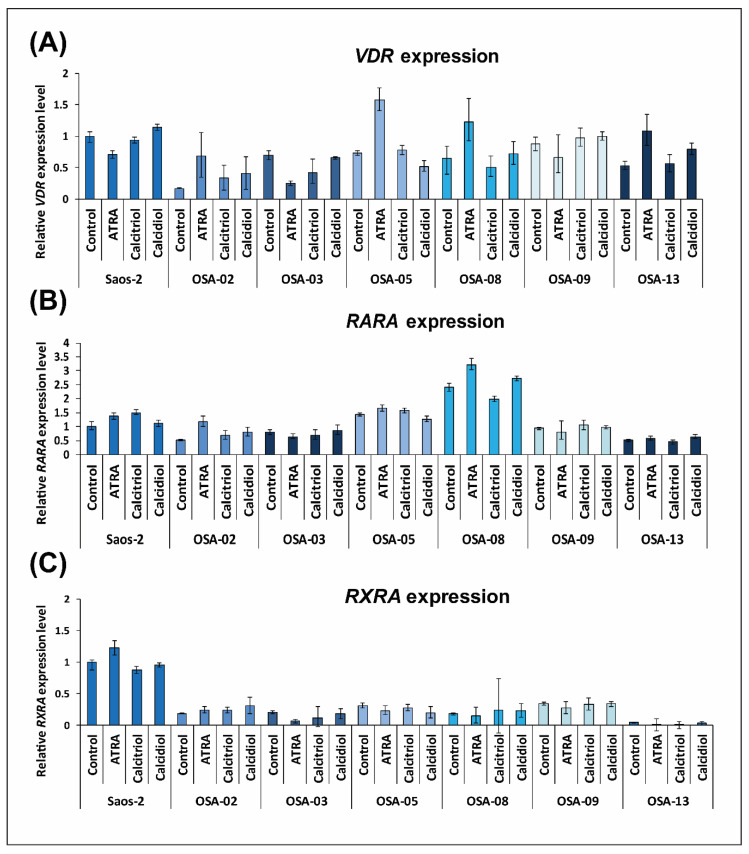
Expression of *RARA*, *RXRA*, and *VDR* mRNA in osteosarcoma cell lines after treatment with selected differentiation inducers. Changes in the expression of the *VDR* (**A**), *RARA* (**B**), and *RXRA* (**C**) genes in osteosarcoma cell lines after 24 h of incubation with 1 µM ATRA, 10 nM calcitriol, or 100 nM calcidiol were measured using RT-qPCR. *GAPDH* served as a loading control. The results are presented as fold changes compared to the mRNA levels detected in untreated control cells. In addition, mRNA levels in untreated patient-derived osteosarcoma cell lines were compared with mRNA levels in the Saos-2 reference cell line, which served as an arbitrary calibrator. Experiments were performed once in technical triplicate. The data represent the mean ± SD.

Based on these results, we focused on changes in the expression of *VDR*, *RARA*, and *RXRA* mRNA after 24 h of experimental treatment with 1 µM ATRA, 10 nM calcitriol, or 100 nM calcidiol. The most interesting result was found for *VDR*, which is apparently upregulated by 1 µM ATRA in patient-derived cell lines (Figure 5A). No apparent trend in *VDR* expression changes was observed after calcitriol and calcidiol exposure. No significant trends in the regulation of *RARA* and *RXRA* expression after treatment with selected drugs were observed (Figure 5B,C).

Given the results of previous analysis, we investigated only VDR expression in detail after ATRA treatment. We treated all selected cell lines with 1 µM ATRA and repeatedly tested the changes at both the mRNA and protein levels.

**Figure 6:**
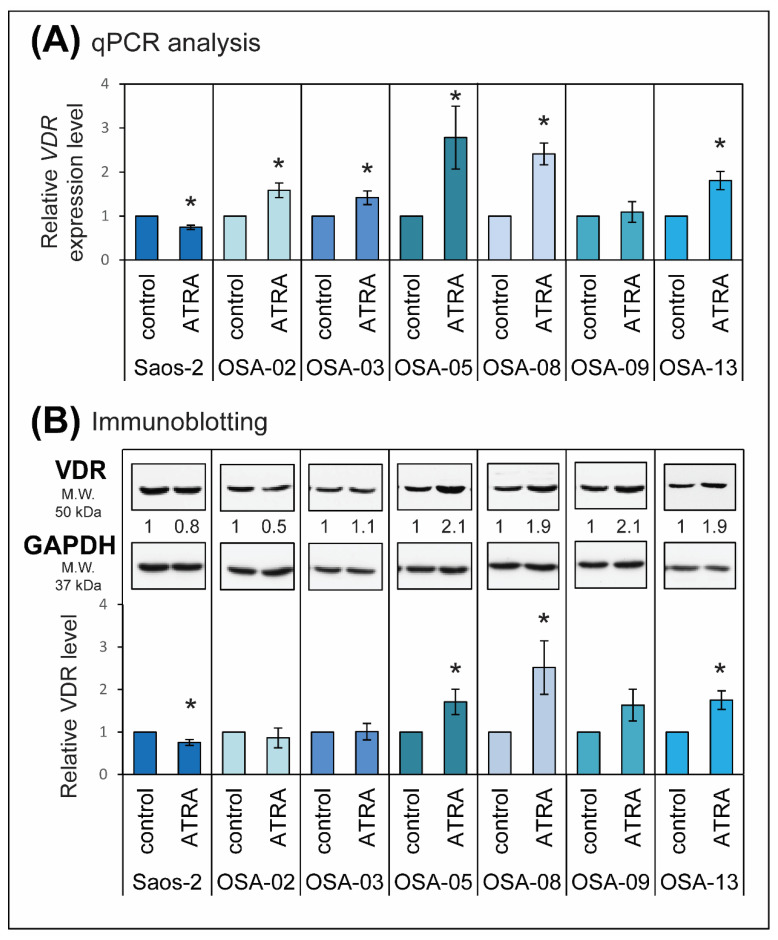
Changes in VDR expression in osteosarcoma cell lines after treatment with ATRA. (**A**) The levels of *VDR* relative gene expression after ATRA treatment were measured using RT-qPCR and are presented as fold changes compared to the levels detected in untreated cells (value = 1), which served as a calibrator (specific for each cell line). The data represent the mean ± SD. Experiments were performed in biological triplicate. (**B**) Levels of VDR protein in osteosarcoma cell lines after 24 h of treatment with 1 µM ATRA were evaluated by immunoblotting. For each cell line, data are presented as VDR level after ATRA treatment compared to the level detected in untreated cells (value = 1), which served as calibrator. GAPDH served as a loading control. The data represent the mean ± SD. Experiments were performed in biological triplicate. Data (**A**,**B**) were statistically analyzed with a one-sample *t*-test (two-tailed): in each selected cell line, values measured in ATRA-treated cells were compared to values in untreated cells (* *p* < 0.05).

On the mRNA level, we observed a downregulation of *VDR* expression by ATRA only in the Saos-2 cell line (Figure 6A). In the OSA-09 cell line, no significant changes were observed after ATRA treatment (Figure 6A). In the remaining cell lines, OSA-02, OSA-03, OSA-05, OSA-08, and OSA-13, ATRA caused a marked increase in *VDR* expression (Figure 6A). On the protein level, we confirmed the changes in VDR levels after ATRA treatment (Figure 6B). An increase in the VDR level was observed in the OSA-05, OSA-08, OSA-09, and OSA-13 cell lines (Figure 6B). No effect of ATRA or a slight decrease in VDR levels was observed in the Saos-2, OSA-02, and OSA-03 cell lines (Figure 6B).

## 3. Discussion

In the present study, we described the responsiveness of seven human osteosarcoma cell lines to two forms of vitamin D_3_ (calcitriol and calcidiol) and to their combinations with the known differentiation inducer ATRA. The Saos-2 established cell line and six patient-derived cell lines were used for experiments.

According to the results from the MTT assay, the Saos-2 established cell line showed only minimal sensitivity to the treatment with calcitriol or calcidiol alone. Although the antiproliferative effect of calcitriol in Saos-2 cell line has already been reported [45,46], another study showed that neither 100 nM calcidiol nor 10 nM calcitriol inhibited proliferative activity in Saos-2 cells after 96 h of treatment [40]. We suspected that this lack of visible inhibition might be due to the early endpoint (96-h), which was not long enough for calcitriol to mediate its downstream action. Therefore, the treatment was extended to 168 h, but no changes in cell proliferation were visible. As the insensitivity of Saos-2 cells was observed in terms of proliferation activity only (i.e., Saos-2 cells were sensitive in terms of induced differentiation) in our experiments, we assume that these inconsistencies may also be caused by different methods of evaluation of the proliferation activity.

In the combination treatments, only calcitriol was able to significantly enhance the inhibitory effect of ATRA. Similarly, we observed that the mRNA level of *BGLAP*, an osteogenic differentiation marker, was highest after combined treatment with calcitriol and ATRA during the entire analyzed period.

The sensitivity of the patient-derived cell lines to differentiation inducers was indeed specific to each cell line. We realized that the increased sensitivity to all differentiation inducers, including calcidiol, in the OSA-13 cell line could be caused by the low differentiation stage of those cells. OSA-13 was previously described as a tumorigenic cell line with elevated expression of the transcriptional factor SOX-2 [47].

Variability in the responsiveness of cell lines could also be related to differences in endogenous levels of respective nuclear receptors for calcitriol and ATRA, as both compounds function as ligands for the respective receptors and subsequently change gene expression [48,49]. We hypothesized that more sensitive cell lines express higher endogenous levels of relevant receptors for these drugs. However, this hypothesis was not confirmed. In general, the expression of respective nuclear receptors in untreated cell lines did not correspond to the inhibition effect of the drugs.

Subsequently, we focused on the evaluation of nuclear receptor expression after 24 h of differentiation inducer treatment and observed that 1 µM ATRA was able to regulate VDR expression. This phenomenon has already been observed in mouse and rat osteosarcoma cell lines [50,51,52,53]. Changes in VDR levels caused by ATRA have already been described in monocytic leukemia cell lines. On the one hand, the majority of research suggests that treatment with ATRA alone is sufficient for VDR regulation [54,55,56,57,58]. On the other hand, one study suggested that only the combined treatment of ATRA and calcitriol effectively increased VDR protein levels but not *VDR* mRNA expression in the THP-1 human monocytic leukemia cell line [59]. In this study, ATRA as a single agent was not able to regulate VDR at the mRNA or protein level [59].

Our results are consistent with the findings described above. Twenty-four hours of treatment with 1 µM ATRA caused changes in *VDR* mRNA levels and VDR protein levels in selected osteosarcoma cell lines. Upregulation or downregulation of VDR depended on the cell line. It was described that there is no *RARE* in the *VDR* promoter, which suggested that ATRA could not regulate *VDR* directly [60]. Therefore, it is assumed that retinoids can regulate *VDR* transcription indirectly using regulatory elements that cooperate with the *VDR* promoter. Moreover, a study on myeloid leukemia cell lines showed that the most important isoform of RAR involved in the regulation of *VDR* transcription is RARα. In the absence of ligands, RARα led to transcriptional repression of the *VDR* gene in this cell type [57].

In accordance with these studies, we focused on the RARα isoform and its agonist ATRA. For better interpretation, we compared the two most different osteosarcoma cell lines—the Saos-2 reference cell line, which had the lowest level of RARα and the highest level of VDR, and the OSA-08 cell line, which had the highest level of RARα and the lowest level of VDR. In the Saos-2 cell line, downregulated expression of VDR was observed after RARα activation by ATRA at both the mRNA and protein levels. In contrast, upregulated expression of VDR was detected in the OSA-08 cell line after ATRA treatment.

Moreover, the combined effect of ATRA and calcitriol was the most effective in the OSA-08 and OSA-13 cell lines, which had high levels of RARα. These data correlate with the hypothesis that unbound RARα acts as a transcriptional repressor of *VDR* [57]. We assume that there is a mechanism involving a change in the RARα conformation after ATRA binding that releases the repression of *VDR* by RARα. After the repression is overcome, cells start to express higher levels of VDR and calcitriol, thus inducing a stronger response. This response even enhances the antineoplastic effect of ATRA, so the combination is more effective than the effect of each drug alone. According to this hypothesis, we expected to see high sensitivity to calcitriol in cell lines with low endogenous expression of RARα, but our experimental data on cell proliferation did not confirm this idea: Saos-2, i.e., the cell line with the lowest endogenous level of RARα, did not respond to calcitriol at any of the used concentrations. In this case, we must take into account that another mechanism of resistance to vitamin D_3_, i.e., an overexpression of VDRE-BP, could be activated in Saos-2 cells [61].

To summarize, our results proved that combination treatment with calcitriol and ATRA showed an enhanced antiproliferative effect compared with the effect of those drugs alone in the majority of tested cell lines. Furthermore, this study provides the first evidence that ATRA treatment influences VDR expression in human osteosarcoma cells in vitro. More specifically, ATRA upregulated VDR expression at the mRNA and protein levels in cell lines with high endogenous levels of RARα and low endogenous levels of VDR; only these cell lines were the most sensitive to the combination treatment. In general, the results suggest that the levels of RARα and VDR in osteosarcoma cells could potentially be used as predictors of possible synergy between calcitriol and ATRA.

## 4. Materials and Methods

### 4.1. Cell Culture

The Saos-2 established cell line (No. HTB-85) was purchased from the American Type Culture Collection (Manassas, VA, USA). Other cell lines were derived from tumor samples obtained during diagnostic biopsies from patients suffering from osteosarcomas. The samples were processed in our laboratory as previously described [62]. The OSA-02, OSA-03, OSA-05, OSA-08, and OSA-13 cell lines were already used and described in our previous studies [47,63,64]. The OSA-09 cell line was derived from the sample of conventional osteosarcoma taken from a 22-year-old patient. The Research Ethics Committee of the School of Medicine (Masaryk University, Brno, Czech Republic) approved the study protocol, and a written statement of informed consent was obtained from each patient or his/her legal guardian prior to participation in this study.

Cells were grown in Dulbecco’s modified Eagle’s medium (DMEM) supplemented with 10% (Saos-2 cells) or 20% (OSA-02, OSA-03, OSA-05, OSA-08, OSA-09, and OSA-13 cells) fetal bovine serum, 100 IU/mL penicillin, 100 mg/mL streptomycin, and 2 mM glutamine (all purchased from GE Healthcare Europe GmbH, Freiburg, Germany). Cell culture was performed under standard conditions at 37 °C in a humidified atmosphere containing 5% CO_2_. Patient-derived cell lines at passages 10–25 were used for the experiments (Supplement 2).

### 4.2. Chemicals

Calcitriol (Sigma-Aldrich, St. Louis, MO, USA) and calcidiol (Sigma-Aldrich) were prepared as stock solutions at a concentration of 1 mM in absolute ethanol (Penta, Prague, Czech Republic) and stored at −20 °C. ATRA (Sigma-Aldrich) was prepared as a stock solution at a concentration of 100 mM in DMSO (Sigma-Aldrich) and stored at −20 °C under light-free conditions. All three stock solutions were freshly diluted in cell culture medium for each use.

### 4.3. Treatment

For proliferation tests, 96-well plates were seeded with 5 × 10^3^ cells per well (Saos-2 cells) or 2 × 10^3^ cells per well (OSA-02, OSA-03, OSA-05, OSA-06, OSA-09, and OSA-13 cells) in 200 μL of complete DMEM. Cells were allowed to adhere overnight. Subsequently, the medium was removed, and fresh medium containing the appropriate concentrations of drugs alone or in combination was added. Cells were treated with five concentrations of calcitriol (10 pM, 100 pM, 1 nM, 10 nM, and 100 nM), five concentrations of calcidiol (100 pM, 1 nM, 10 nM, 100 nM, and 1 μM), and one concentration of ATRA (1 μM). The plates were incubated under standard conditions for 3 or 7 days.

To prepare samples for immunoblotting and PCR analyses, cells were seeded onto Petri dishes and allowed to adhere overnight. The medium was removed and replaced with fresh medium containing 10 nM calcitriol, 100 nM calcidiol, and/or 1 μM ATRA. For immunoblotting and qPCR, cells were harvested after 24 h of treatment, and for semiquantitative RT-PCR, cells were harvested after 1, 3, 5, 7, and 9 days of treatment.

In all experiments, untreated cells were used as controls. In addition, we compared the proliferation activity of untreated cells and cells treated with vehicle (DMSO/ethanol) only and found no significant difference.

### 4.4. Cell Viability

Cell viability was evaluated using the MTT assay, which was performed as previously described [25]. Briefly, the plates with 0.5 mg/mL 3-[4,5-dimethylthiazol-2-yl]-2,5-diphenyl- tetrazolium bromide (MTT) (Sigma-Aldrich) were incubated at 37 °C for 3 hours. Formazan crystals were dissolved in 200 μL of DMSO. The absorbance at 570 nm was measured with a reference absorbance at 620 nm using a Sunrise Absorbance Reader (Tecan, Männedorf, Switzerland). Each experiment was performed in triplicate. The results obtained were expressed as a percentage of untreated controls.

### 4.5. RT-PCR

The expression of osteogenic differentiation markers was evaluated using semiquantitative RT-PCR. The protocol included standard procedures that were previously described [15]. The primers for genes of interest are listed in Table 2. The optical density of bands was quantified using ImageJ software, and the data were normalized to *HSP90AB1* expression. Each experiment was performed in triplicate.

The relative expression levels of selected nuclear receptors were studied using RT-qPCR. Total RNA was extracted and reverse transcribed into cDNA in the same manner as described previously [25]. RT-qPCR was carried out in 10 μL using the KAPA SYBR^®^ FAST qPCR Kit (Kapa Biosystems, Wilmington, MA, USA) and analyzed using the 7500 Fast Real-Time PCR System and 7500 Software v. 2.0.6 (both Life Technologies, Carlsbad, CA, USA). Changes in the transcript levels were calculated using Cq values standardized to a housekeeping gene (*GAPDH*) used as an endogenous reference gene control. The established Saos-2 cell line served as the arbitrary calibrator. The primers used for genes of interest are provided in Table 5. Each experiment was performed in triplicate.

### 4.6. Immunoblotting

Cells were lysed in LB1 buffer (50 mM Hepes-KOH, pH 7.5, 140 mM NaCl, 1 mM EDTA, 10% glycerol, 0.5% NP-40, 0.25% Triton X-100), and the total protein amount was subsequently measured by the DC Protein Arrays Reagents Package (Bio-Rad Laboratories, Munich, Germany) according to the manufacturer’s instructions. Total proteins (10 μg) were loaded onto 10% polyacrylamide gels, electrophoresed, and blotted on a polyvinylidene difluoride membrane (Bio-Rad Laboratories). The membranes were blocked with 5% nonfat dry milk in PBS with 0.1% Tween-20 (Sigma-Aldrich) and incubated with primary antibodies overnight. The next day, membranes were incubated with secondary antibodies at room temperature (RT) for 1 hour. All antibodies used for immunoblotting are listed in Table 6. ECL-Plus detection was performed according to the manufacturer’s instructions (GE Healthcare). The optical density of bands was quantified using ImageJ software, and the data were normalized to loading control GAPDH. Each experiment was performed in triplicate.

### 4.7. Statistics

Quantitative data were statistically evaluated using SPSS Statistics software (version 25.0, IBM, New York, USA). Data obtained in the MTT assay were analyzed by one-way ANOVA, followed by the Scheffé post hoc test: * *p* < 0.05 and ** *p* < 0.001 were considered statistically significant. Analysis of possible interactions of compounds included in this study was performed using the Bliss independence model [65]. Data obtained using PCR and immunoblotting were analyzed with a one-sample *t*-test (two-tailed): * *p* < 0.05 was considered statistically significant.

## Figures and Tables

**Table 1:** Analysis of possible interactions of individual compounds in Saos-2 established cell line using Bliss independence model. Detailed calculations are provided in Table A1 and Table A2.

Combination of Drugs		Saos-2 Cells		Combination of Drugs		Saos-2 Cells	
Calcitriol	ATRA	Day 3	Day 7	Calcidiol	ATRA	Day 3	Day 7
0.01 nM	1 µM			0.1 nM	1 µM		
0.1 nM	1 µM			1 nM	1 µM		
1 nM	1 µM			10 nM	1 µM		
10 nM	1 µM			100 nM	1 µM		
100 nM	1 µM			1000 nM	1 µM		

Color legend: green, synergy; blue, additive effect; red, antagonism.

**Table 2:** Comparison of the effects of drug combinations with the sum of the effects of individual drugs in RT-PCR experiments with Saos-2 cell line.

	Day 1	Day 3	Day 5	Day 7	Day 9
**Up-regulation of *BGLAP* expression after combined treatment with ATRA and calcitriol [%]**					
Predicted effect	**0.62**	**0.42**	**1.10**	**1.43**	**1.23**
Observed effect	**1.26**	**0.60**	**0.91**	**1.50**	**1.05**
**Up-regulation of *BGLAP* expression after combined treatment with ATRA and calcidiol [%]**					
Predicted effect	**0.38**	**0.18**	**0.64**	**0.71**	**0.60**
Observed effect	**0.65**	**0.07**	**0.59**	**0.71**	**0.37**
**Up-regulation of *SPP-1* expression after combined treatment with ATRA and calcitriol [%]**					
Predicted effect	**1.05**	**0.83**	**3.66**	**1.86**	**2.13**
Observed effect	**0.70**	**0.24**	**3.61**	**1.41**	**1.38**
**Up-regulation of *SPP-1* expression after combined treatment with ATRA and calcidiol [%]**					
Predicted effect	**0.59**	**0.58**	**3.23**	**1.89**	**1.87**
Observed effect	**0.49**	**0.19**	**3.65**	**1.76**	**1.63**

Color legend: green, synergy; blue, additive effect; red, antagonism.

**Table 3:** Analysis of possible interactions of calcitriol and ATRA in patient-derived cell lines using Bliss independence model. Detailed calculations are provided in Table A1.

Concentrations of Drugs		Patient-Derived Cell Lines					
Calcitriol	ATRA	OSA-02	OSA-03	OSA-05	OSA-08	OSA-09	OSA-13
0.01 nM	1 µM						
0.1 nM	1 µM						
1 nM	1 µM						
10 nM	1 µM						
100 nM	1 µM						

Color legend: green, synergy; blue, additive effect; red, antagonism.

**Table 4:** Analysis of possible interactions of calcidiol and ATRA in patient-derived cell lines using Bliss independence model. Detailed calculations are provided in Table A2.

Concentrations of Drugs		Patient-Derived Cell Lines					
Calcidiol	ATRA	OSA-02	OSA-03	OSA-05	OSA-08	OSA-09	OSA-13
0.1 nM	1 µM						
1 nM	1 µM						
10 nM	1 µM						
100 nM	1 µM						
1000 nM	1 µM						

Color legend: green, synergy; blue, additive effect; red, antagonism.

**Table 5:** Sequences of primers used for RT-PCR.

Gene	Primer Sequence	Product Length (bp)
*BGLAP*	F: 5′-GAG GGC AGC GAG GTA GTG AA-3′	152
	R: 5′-TCC TGA AAG CCG ATG TGG TC-3′	
*SPP1*	F: 5′-GCC GAG GTG ATA GTG TGG TT-3′	242
	R: 5′-GTG GGT TTC AGC ACT CTG GT-3′	
*HSP90AB1*	F: 5′-CGC ATG AAG GAG ACA CAG AA-3′	169
	R: 5′-TCC CAT CAA ATT CCT TGA GC-3′	
*RARA*	F: 5’-CGA CCG AAA CAA GAA GAA GAA GG-3´	166
	R: 5´-TTC TGA GCT GTT GTT CGT AGT GT-3´	
*RXRA*	F: 5´-CTC AAT GGC GTC CTC AAG GT-3´	111
	R: 5´-CAC TCC ATA GTG CTT GCC TGA-3´	
*VDR*	F: 5´-AGC CTC AAT GAG GAG CAC TCC AAG-3´	206
	R: 5´-ACG GGT GAG GAG GGC TGC TGA GTA-3´	
*GAPDH*	F: 5´-AGC CAC ATC GCT CAG ACA CC-3´	302
	R: 5´-GTA CTC AGC GCC AGC ATC G-3´	

F, forward primer; R, reverse primer.

**Table 6:** Primary and secondary antibodies.

**Primary Antibodies**					
**Antigen**	**Type/Host**	**Clone**	**Catalog No.**	**Manufacturer**	**Dilution**
RARα	Mono/Mo	H1920	ab41934	Abcam	1:1000
RXRα	Mono/Rb	D6H10	3085	Cell Signaling	1:1000
VDR	Mono/Rb	EPR4552	ab109234	Abcam	1: 2000
GAPDH	Mono/Rb	14C10	2118S	Cell Signaling	1:10,000
**Secondary Antibodies**					
**Host**	**Specificity**	**Conjugate**	**Catalog No.**	**Manufacturer**	**Dilution**
Goat	anti-Rb IgG	HRP	7074	Cell Signaling	1:5000
Horse	anti-Mo IgG	HRP	7076	Cell Signaling	1:5000

Type: Mono, monoclonal. Host: Rb, rabbit; Mo, mouse.

## Supplementary Materials

Supplementary materials can be found at https://www.mdpi.com/1422-0067/21/18/6591/s1.

## Abbreviations

ATRA	All-*trans* retinoic acid
COX	Cyclooxygenase
DMEM	Dulbecco’s modified Eagle’s medium
DMSO	Dimethyl sulfoxide
GAPDH	Glyceraldehyde-3-phosphate dehydrogenase
LOX	Lipoxygenase
MTT	3-[4,5-dimethylthiazol-2-yl]-2,5-diphenyltetrazolium bromide
RARα	Retinoic acid receptor α
RARE	Retinoic acid response element
RXRα	Retinoid X receptor α
VDR	Vitamin D receptor
VDRE	Vitamin D response element
VDRE-BP	Vitamin D response element-binding protein

## Appendix A

**Table A1:** Computational analysis of calcitriol and ATRA interactions using Bliss independence model.

	Calcitriol (*Y_a_*)		ATRA 1 µM (*Y_b_*)	*Y_ab,P_*	*Y* *_ab,O_*		Calcitriol (*Y_a_*)		ATRA 1 µM (*Y_b_*)	*Y_ab,P_*	*Y* *_ab,O_*
**Saos-2** **Day 3**	0.01 nM	0.03	0.27	0.29	**0.35**	**OSA-05**	0.01 nM	−0.01	−0.12	−0.13	**0.03**
	0.1 nM	−0.01	0.27	0.26	**0.33**		0.1 nM	0.04	−0.12	−0.07	**0.04**
	1 nM	0.03	0.27	0.29	**0.33**		1 nM	−0.06	−0.12	−0.19	**−0.04**
	10 nM	0.05	0.27	0.30	**0.40**		10 nM	0.13	−0.12	0.02	**−0.03**
	100 nM	0.03	0.27	0.28	**0.38**		100 nM	0.13	−0.12	0.02	**0.02**
**Saos-2** **Day 7**	0.01 nM	−0.01	0.35	0.34	**0.48**	**OSA-08**	0.01 nM	0.13	0.28	0.37	**0.43**
	0.1 nM	−0.03	0.35	0.34	**0.48**		0.1 nM	0.18	0.28	0.41	**0.41**
	1 nM	0.00	0.35	0.35	**0.47**		1 nM	0.16	0.28	0.39	**0.41**
	10 nM	0.03	0.35	0.37	**0.49**		10 nM	0.21	0.28	0.43	**0.42**
	100 nM	0.02	0.35	0.37	**0.48**		100 nM	0.36	0.28	0.54	**0.48**
**OSA-02**	0.01 nM	0.05	0.20	0.24	**0.27**	**OSA-09**	0.01 nM	−0.02	0.02	0.00	**−0.04**
	0.1 nM	0.17	0.20	0.33	**0.25**		0.1 nM	−0.03	0.02	−0.01	**−0.07**
	1 nM	0.20	0.20	0.36	**0.32**		1 nM	0.18	0.02	0.19	**−0.11**
	10 nM	0.23	0.20	0.38	**0.37**		10 nM	0.25	0.02	0.26	**−0.15**
	100 nM	0.51	0.20	0.60	**0.47**		100 nM	0.38	0.02	0.38	**0.00**
**OSA-03**	0.01 nM	0.03	−0.15	−0.12	**−0.06**	**OSA-13**	0.01 nM	0.07	0.35	0.40	**0.43**
	0.1 nM	0.03	−0.15	−0.12	**−0.09**		0.1 nM	0.12	0.35	0.43	**0.46**
	1 nM	0.08	−0.15	−0.06	**−0.04**		1 nM	0.16	0.35	0.45	**0.45**
	10 nM	0.14	−0.15	0.01	**0.03**		10 nM	0.20	0.35	0.48	**0.49**
	100 nM	0.41	−0.15	0.32	**0.26**		100 nM	0.39	0.35	0.60	**0.54**

*Y_a_*, inhibition rate (%) of drug A (calcitriol) alone at dose *a*; *Y_b_*, inhibition rate (%) of drug B (ATRA) alone at dose *b*; *Y_ab,P_*, Bliss-predicted inhibition rate calculated as *Y_ab,P_* = *Y_a_* + *Y_b_* – *Y_a_* * *Y_b_*; *Y_ab,O_*, observed inhibition rate at combination dose (*a* + *b*) of drug A and drug B; *Y_ab,O_* > *Y_ab,P_*, synergy (green); *Y_ab,O_* = *Y_ab,P_*, additive effect (blue); *Y_ab,O_* < *Y_ab,P_*, antagonism (red).

**Table A2:** Computational analysis of calcidiol and ATRA interactions using Bliss independence model.

	Calcidiol (*Y_a_*)		ATRA 1 µM (*Y_b_*)	*Y_ab,P_*	*Y* *_ab,O_*		Calcidiol (*Y_a_*)		ATRA 1 µM (*Y_b_*)	*Y_ab,P_*	*Y* *_ab,O_*
**Saos-2** **Day 3**	0.1 nM	−0.02	0.27	0.25	**0.30**	**OSA-05**	0.1 nM	0.07	−0.12	−0.05	**0.04**
	1 nM	0.01	0.27	0.28	**0.29**		1 nM	0.07	−0.12	−0.05	**0.03**
	10 nM	0.04	0.27	0.29	**0.31**		10 nM	0.08	−0.12	−0.03	**0.01**
	100 nM	0.03	0.27	0.29	**0.32**		100 nM	0.03	−0.12	−0.09	**−0.07**
	1000 nM	−0.03	0.27	0.24	**0.28**		1000 nM	0.29	−0.12	0.21	**0.12**
**Saos-2** **Day 7**	0.1 nM	0.03	0.35	0.37	**0.24**	**OSA-08**	0.1 nM	0.05	0.28	0.31	**0.40**
	1 nM	0.04	0.35	0.38	**0.26**		1 nM	0.09	0.28	0.34	**0.40**
	10 nM	0.03	0.35	0.37	**0.26**		10 nM	0.10	0.28	0.35	**0.39**
	100 nM	0.03	0.35	0.37	**0.26**		100 nM	0.09	0.28	0.34	**0.37**
	1000 nM	0.08	0.35	0.40	**0.29**		1000 nM	0.32	0.28	0.51	**0.44**
**OSA-02**	0.1 nM	0.00	0.20	0.20	**0.28**	**OSA-09**	0.1 nM	−0.01	0.02	0.01	**−0.08**
	1 nM	0.13	0.20	0.30	**0.25**		1 nM	0.01	0.02	0.02	**−0.06**
	10 nM	0.10	0.20	0.28	**0.26**		10 nM	0.03	0.02	0.04	**−0.06**
	100 nM	0.12	0.20	0.30	**0.21**		100 nM	0.07	0.02	0.09	**−0.12**
	1000 nM	0.47	0.20	0.57	**0.29**		1000 nM	0.30	0.02	0.31	**0.00**
**OSA-03**	0.1 nM	−0.02	−0.15	−0.18	**−0.10**	**OSA-13**	0.1 nM	0.09	0.35	0.41	**0.48**
	1 nM	0.02	−0.15	−0.13	**−0.16**		1 nM	0.16	0.35	0.46	**0.49**
	10 nM	−0.01	−0.15	−0.16	**−0.10**		10 nM	0.19	0.35	0.47	**0.50**
	100 nM	0.00	−0.15	−0.16	**−0.13**		100 nM	0.21	0.35	0.49	**0.50**
	1000 nM	0.42	−0.15	0.33	**0.31**		1000 nM	0.44	0.35	0.64	**0.55**

*Y_a_*, inhibition rate (%) of drug A (calcidiol) alone at dose *a*; *Y_b_*, inhibition rate (%) of drug B (ATRA) alone at dose *b*; *Y_ab,p_*, Bliss-predicted inhibition rate calculated as *Y_ab,P_* = *Y_a_* + *Y_b_* − *Y_a_* * *Y_b_*; *Y_ab,o_*, observed inhibition rate at combination dose (*a* + *b*) of drug A and drug B; *Y_ab,O_* > *Y_ab,p_*, synergy (green); *Y_ab,O_* = *Y_ab,p_*, additive effect (blue); *Y_ab,O_* < *Y_ab,p_*, antagonism (red).

**Table A3:** Statistical analysis of relative expression of *BGLAP* and *SPP1* in Saos-2 cell line.

***BGLAP* Relative Expression**						
**DAYS**		**ATRA**	**CALCITRIOL**	**ATRA + CALCITRIOL**	**CALCIDIOL**	**ATRA + CALCIDIOL**
1	MEAN	1.198	1.426	2.255	1.185	1.645
	SD	0.027	0.040	0.230	0.168	0.882
	STATISTICS	*	*	*		
3	MEAN	1.292	1.124	1.604	0.888	1.067
	SD	0.094	0.220	0.320	0.085	0.220
	STATISTICS	*				
5	MEAN	1.633	1.472	1.908	1.010	1.587
	SD	0.182	0.344	0.370	0.208	0.384
	STATISTICS	*		*		
7	MEAN	1.749	1.681	2.497	0.964	1.710
	SD	0.218	0.325	0.680	0.182	0.494
	STATISTICS	*	*	*		
9	MEAN	1.728	1.505	2.054	1.710	1.366
	SD	0.430	0.244	0.022	0.108	0.464
	STATISTICS		*	*		
***SPP1* Relative Expression**						
**DAYS**		**ATRA**	**CALCITRIOL**	**ATRA + CALCITRIOL**	**CALCIDIOL**	**ATRA + CALCIDIOL**
1	MEAN	1.619	1.432	1.705	0.972	1.495
	SD	0.284	0.167	0.383	0.223	0.341
	STATISTICS					
3	MEAN	1.828	1.000	1.238	0.754	1.191
	SD	0.182	0.351	0.097	0.188	0.422
	STATISTICS	*				
5	MEAN	4.181	1.481	4.613	1.047	4.646
	SD	0.506	0.247	0.192	0.198	0.787
	STATISTICS	*		*		*
7	MEAN	2.730	1.132	2.411	1.163	2.760
	SD	0.354	0.067	0.631	0.038	0.794
	STATISTICS	*		*		*
9	MEAN	3.022	1.105	2.378	0.850	2.634
	SD	0.575	0.412	0.771	0.174	0.820
	STATISTICS	*				*

The data were analyzed by one-sample *t*-test (two-tailed); * *p* < 0.05 was consider statistically significant. Experiments were performed in biological triplicate.
