# Supplementary material for: Enhanced Antiproliferative Effect of Combined Treatment with Calcitriol and All-Trans Retinoic Acid in Relation to Vitamin D Receptor and Retinoic Acid Receptor α Expression in Osteosarcoma Cell Lines
Source: Int J Mol Sci. 2020 Sep 9;21(18):6591. doi: 10.3390/ijms21186591 (PMC7554701; doi:10.3390/ijms21186591)
Supplement: Supplementary file 1 [file ijms-21-06591-s001.pdf]

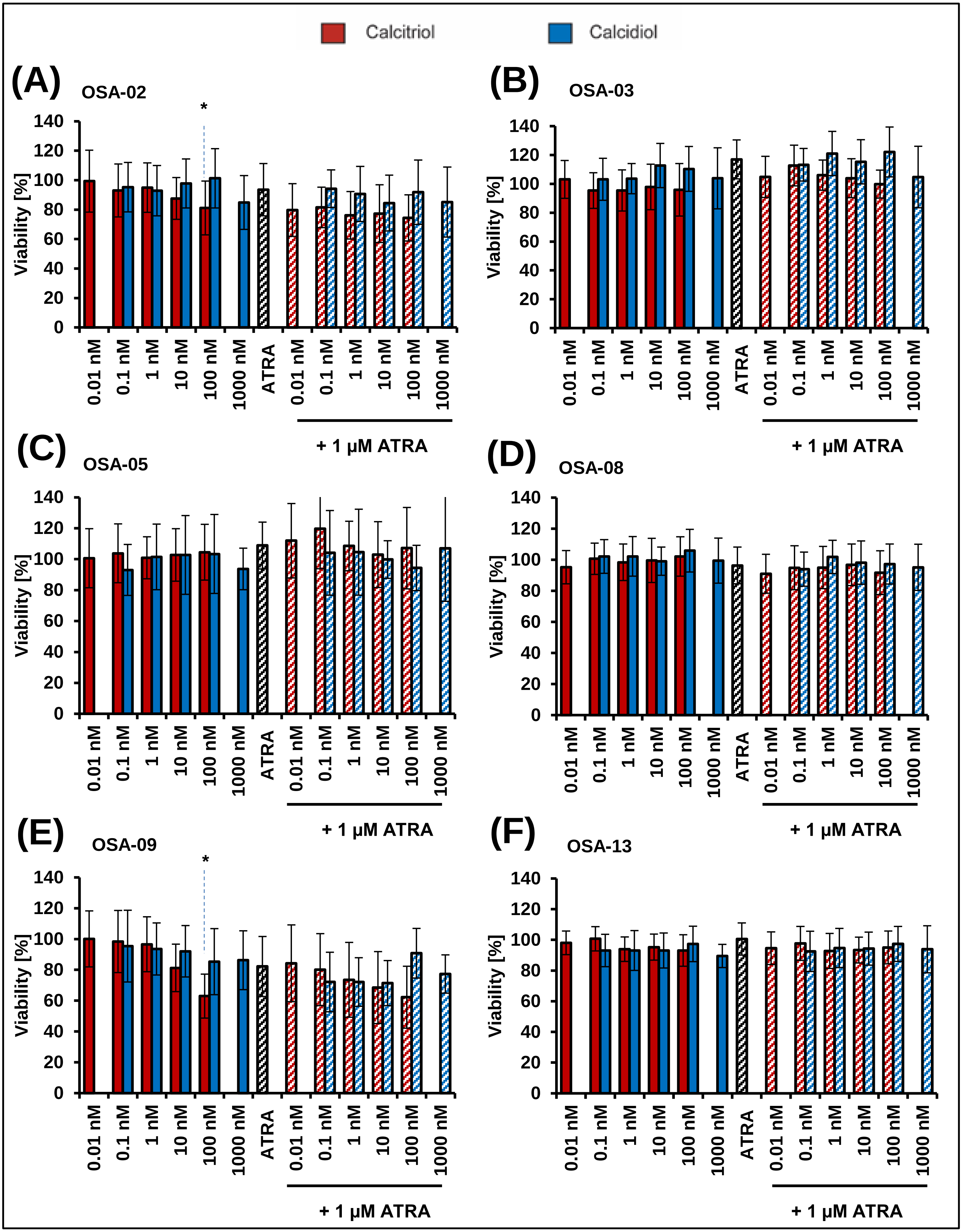

Supplement 1: Proliferation of patient-derived osteosarcoma cell lines after 3 days of selected treatment. The proliferation of the OSA-02 **(A)**, OSA-03 **(B)**, OSA-05 **(C)**, OSA-08 **(D)**, OSA-09 **(E)**, and OSA-13 **(F)** cell lines was measured using the MTT assay on day 3 of incubation with various concentrations of calcitriol or calcidiol alone, 1 µM ATRA alone or drug combinations. The values were compared with those in untreated cells, whose proliferation activity was set as 100%. The data represent the mean ± SD. The results were analyzed using one-way ANOVA followed by the Scheffé post hoc test. The proliferation of cells treated with calcitriol or calcidiol alone was compared to the proliferation of untreated control cells: * $P < 0.05$, ** $P < 0.001$. The proliferation of cells treated with a combination of drugs was compared to the proliferation of cells treated with ATRA alone: $\Delta < 0.05$, $\Delta\Delta < 0.001$. Experiments were performed in biological triplicates.

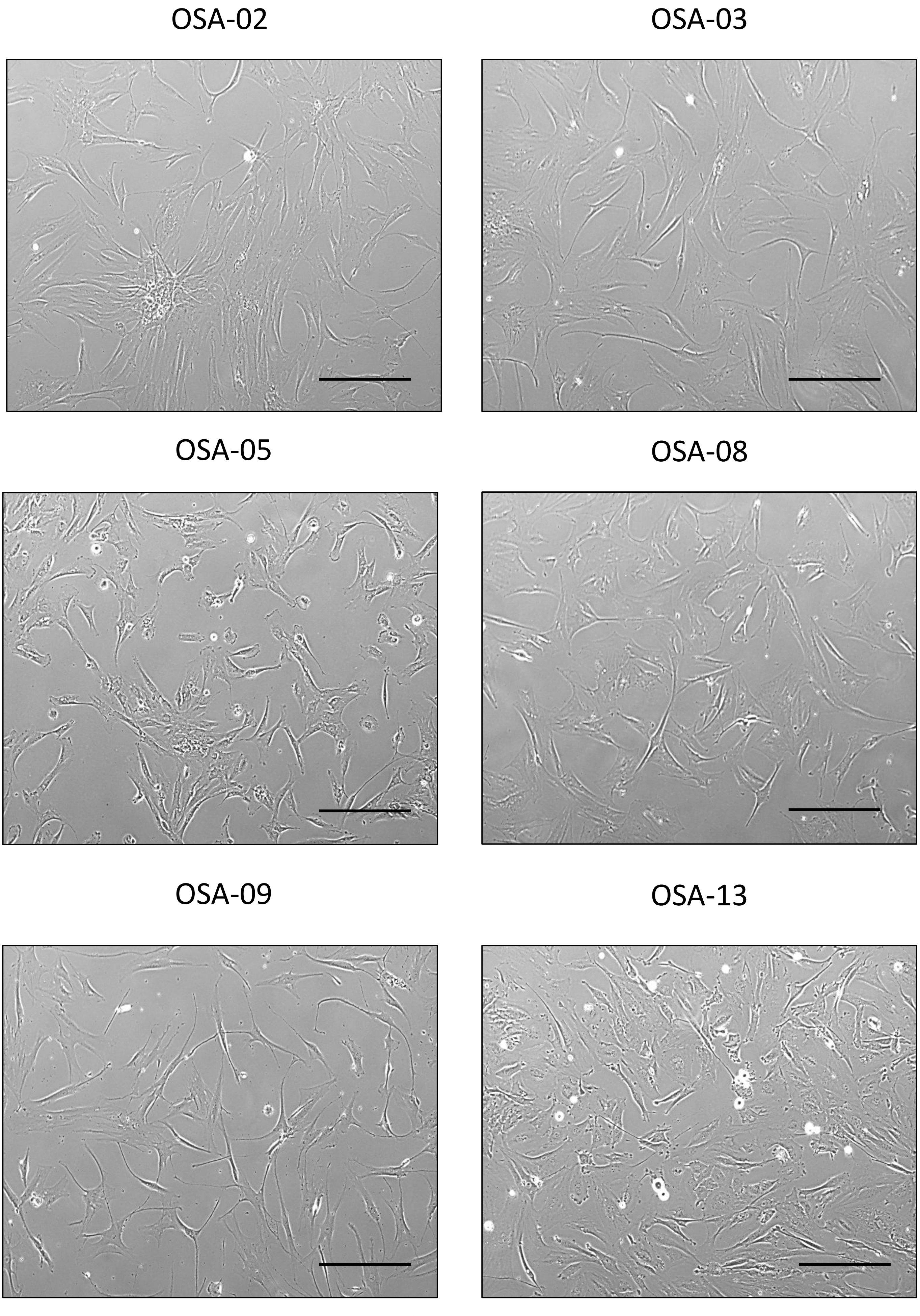

Supplement 2: The untreated patient-derived cell lines. Scale bars show 200 μm.
